# Quantifying Dehydration Effects of Porcine Vocal Fold Attenuation With Optical Coherence Tomography

**DOI:** 10.1002/lary.70529

**Published:** 2026-03-26

**Authors:** Owen P. Wischhoff, Michael Kuang, Brady D. Prosser, Jakob R. Holm, Jack J. Jiang

**Affiliations:** ^1^ Department of Otolaryngology–Head and Neck Surgery University of Wisconsin School of Medicine and Public Health Madison WI USA

**Keywords:** dehydration, edema, ex vivo, optical coherence tomography (OCT), vocal fold (VF)

## Abstract

**Objective:**

The purpose of this study is to use optical coherence tomography (OCT) to characterize the effect of dehydration on the optical attenuation coefficient of porcine vocal fold (VF) tissue. Assessing laryngeal hydration is clinically relevant for evaluating vocal function; however, no reliable noninvasive methods currently exist to quantify it.

**Methods:**

Six porcine larynges were bisected into 12 halves, yielding 12 VFs. Each specimen underwent serial dehydration using a vacuum oven to approximately 5%, 10%, and 15% mass loss, with OCT scans obtained at each interval using a 1300 nm wavelength system. Attenuation coefficients were extracted from OCT B‐scans through custom MATLAB processing, and the relationship between tissue dehydration and attenuation was modeled using a linear mixed‐effects model.

**Results:**

Across VFs, attenuation decreased by approximately 0.08 mm^−1^ for each 1% increase in tissue mass loss (*b* = −0.079, 95% CI [−0.105, −0.054]). Attenuation decreased linearly with increasing tissue dehydration. The true VF exhibited consistently higher attenuation values than the false folds by an average of 1.47 mm^−1^ (*p* < 0.001). Interfold variability was low (ICC = 0.041), indicating strong reproducibility across specimens.

**Conclusions:**

OCT‐derived attenuation provides a quantitative, reproducible indicator of VF dehydration. The linear relationship between attenuation and tissue mass loss supports the feasibility of OCT as a noninvasive optical biomarker of VF hydration. Future multi‐wavelength and in vivo studies will be critical to translate this approach into clinical monitoring of VF hydration, edema, and lesion formation.

**Level of Evidence:**

NA

## Graphical abstract

The purpose of this study is to use optical coherence tomography (OCT) to characterize the effect of dehydration on the optical attenuation coefficient of porcine vocal fold (VF) tissue. The optical attenuation coefficient tissue decreased linearly with dehydration, dropping by approximately 0.08 mm^−1^ for every 1% increase in mass loss. These results suggest that OCT could serve as a noninvasive, quantitative biomarker for monitoring laryngeal hydration and edema in clinical settings.

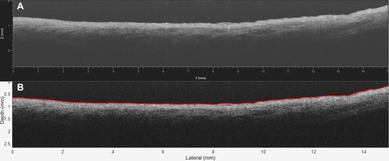

## 1 Introduction

Roughly one in five Americans (20.6%) report having experienced a voice disorder at some point in their lives, with these rates increasing [1, 2]. Optimal vocal fold (VF) function depends on the biomechanical properties of the VF lamina propria, which the biphasic theory describes as a viscoelastic system governed by stress‐relaxation behavior [3]. As the intermediate layer of the VF, the lamina propria is composed of both solid and liquid components, reflecting its biphasic nature [4]. Adequate hydration is essential to maintain the viscoelastic behavior of the VF lamina propria, as dehydration increases tissue viscosity and adversely affects voice production [5]. During phonation onset, stress is initially concentrated in the solid component of the VF tissue but decreases as fluid redistributes within the lamina propria. Dehydration disrupts this process, elevating tissue stress and increasing susceptibility to VF lesions such as nodules, polyps, and Reinke's edema [4], which can affect social outcomes and work performance [6].

Although hydration is critical for normal vocal function, there are no reliable non‐invasive techniques for quantifying VF hydration currently [4, 7, 8]. Current clinical imaging techniques are restricted to surface visualization of the VFs, with submucosal characteristics inferred indirectly from stroboscopic or high‐speed video analyses [9]. However, optical coherence tomography (OCT) offers a clinically viable, noninvasive imaging modality capable of precise cross‐sectional imaging of tissue internal structures [8, 9, 10]. OCT has historically been used in ophthalmology as a noninvasive method to diagnose ocular pathologies, such as glaucoma, macular degeneration, and diabetic retinopathy [11]; however, its potential application in disciplines outside of ophthalmology, such as dermatology [12], pulmonology [13], and otolaryngology [10, 14, 15, 16, 17, 18, 19], is evident. Coughlan et al. used Doppler OCT imaging in vivo to characterize VF lesions using depth invasion as a proxy [9]. Burns et al. demonstrated that OCT can be applied in vivo to confirm subepithelial placement of hydrogel implants and to characterize VF lesions, particularly those involving scar tissue [16, 17].

While prior OCT studies have primarily focused on structural imaging and lesion characterization [9, 14, 17, 19, 20, 21, 22], the same imaging capabilities also provide an opportunity to evaluate tissue hydration. Because hydration plays a central role in maintaining the viscoelastic properties necessary for normal vocal function and in preventing benign lesions [3], developing an OCT‐based assessment of subepithelial water content would represent a promising clinical advancement. OCT has been used numerous times clinically [9, 16, 17], demonstrating its feasibility as an in vivo imaging modality. However, its potential for quantifying VF hydration has not yet been explored.

OCT operates by splitting near‐infrared light into a reference beam and a sample beam [23]. The sample beam penetrates the tissue and decays with depth, while the reference beam travels a known optical path; interference between the two beams at the detector produces a depth‐resolved profile (A‐scan) of the tissue. Multiple A‐scans are combined laterally to generate a two‐dimensional cross‐section, or B‐scan [23, 24]. The intensity of the sample beam decays with depth due to scattering and absorption, a phenomenon quantified by the optical attenuation coefficient (*μ*_OCT_). Absorption and scattering each describe the probability of sample beam light decay per unit path length, and their combined effects define the total attenuation of light in tissue [25]. The scattering coefficient (*μ*_s_) results from gradients in the tissues' refractive index, redirecting light by refraction. These refractive‐index gradients originate from subcellular and extracellular structures, such as collagen fibers, membranes, proteins, and organelles [25, 26]. Alternatively, absorption is wavelength‐dependent and reduces light intensity by converting it into other forms of energy. The absorption coefficient (*μ*_a_) is determined by the concentration of tissue chromophores, with the dominant absorbers at infrared wavelengths being hemoglobin, melanin, and water [25]. Ultimately, this study aims to determine how attenuation changes systematically with progressive tissue dehydration, assess whether this relationship differs between the true and false VFs, and evaluate the reproducibility of these changes across specimens.

It is hypothesized that the attenuation coefficient would decrease linearly with increasing tissue dehydration. Since water is a dominant absorber in the near‐infrared spectrum, decreases in tissue hydration should lower absorption and, subsequently, total attenuation. Furthermore, it was expected that interspecimen variability would be minimal, supporting the reproducibility and robustness of OCT‐based attenuation as a quantitative marker of tissue hydration. By establishing this quantitative correlation between hydration and OCT‐derived attenuation, this study provides foundational evidence for the utilization of OCT as a noninvasive biomarker of VF hydration and tissue condition.

## 2 Materials and Methods

### 2.1 Tissue Sample Preparation

Pigs were ethically euthanized for purposes unrelated to this study and purchased from the Animal & Dairy Science Department at the University of Wisconsin–Madison. After harvesting, the larynges were frozen in 0.9% saline solution stored at −12°C for approximately 2 weeks before data collection. Before data collection, the larynges were thawed in a water bath and heated to room temperature. Six fresh porcine larynges were bisected into 12 halves, yielding 12 VFs. All guidelines provided by the University of Wisconsin–Madison Institutional Animal Care and Use Committee were followed. As such, the prior approval of tissue use is not required if the animal has already been euthanized.

### 2.2 Equipment and Data Collection Protocol

**FIGURE 1:**
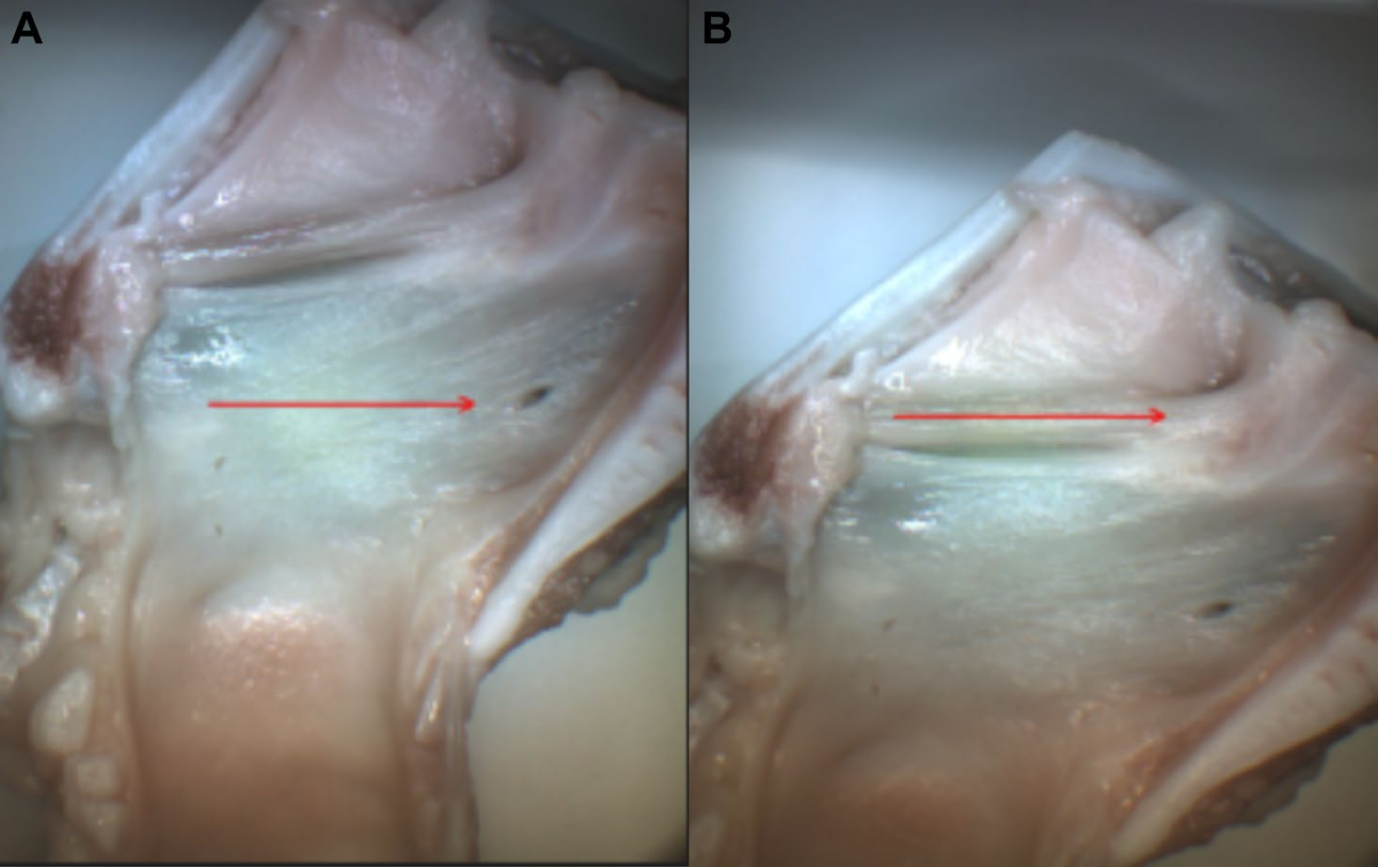
Image of optical coherence tomography (OCT) scan for (A) Vocal fold 3 half one (VF3H1) midline control. (B) VF3H1 false fold control. The red arrow indicates scan direction. [Color figure can be viewed in the online issue, which is available at www.laryngoscope.com]

All 12 larynx tissues were weighed at room temperature and scanned initially on a Thorolabs Telesto‐II OCT machine. Then, a series of dehydrations and subsequent scans was performed so that the larynges lost roughly 5%, 10%, and 15% of their initial mass and were scanned after each dehydration mark. Dehydration time was consistent across larynges, which ranged from 10 to 45 min. Dehydration was accomplished by vacuum desiccation in a vacuum oven at −25 inHg (0.165 atm) and 55°C until the desired dehydration level was achieved. The larynges were positioned near the bottom of the oven, where the temperature was lower than the set point, resulting in slower dehydration. Since dehydration was treated as a continuous variable, these time points represent approximate intervals chosen to ensure consistent exposure durations across samples. The OCT machine used a wavelength of 1300 nm. A‐scans were averaged every 20 scans (A‐scan averaging = 20) and used to construct the B‐scan. The scan width was 15 mm (1000 pixels, 15 μm/pixel), and the scan depth was 2.65 mm (1024 pixels, 2.59 μm/pixel). Only one B‐scan was taken; two scans were conducted on each larynx, one on the vestibular VFs and another on the midline of the VF for consistency and high signal quality (Figure 1). The scan orientation was consistent across varying hydration levels, achieved by creating a punctured hole and scanning along the orientation parallel to the false VF to minimize regional microstructural variability. Between scans, tissues were stored in sealed containers to prevent uncontrolled changes in hydration. The scans were exported as .oct files using the Thorolabs Viewer software.

### 2.3 Image Processing

OCT B‐scans were processed using an in‐house MATLAB script. Unaltered OCT scans were exported as a single page. TIFF files were using Thorolabs Viewer and converted to grayscale in MATLAB before analysis. The tissue surface was identified with an adaptive detector requiring:

- the local intensity to exceed a threshold estimated from the background noise of the air band
- the gradient magnitude to exceed a fraction of the selected column's maximum.

**FIGURE 2:**
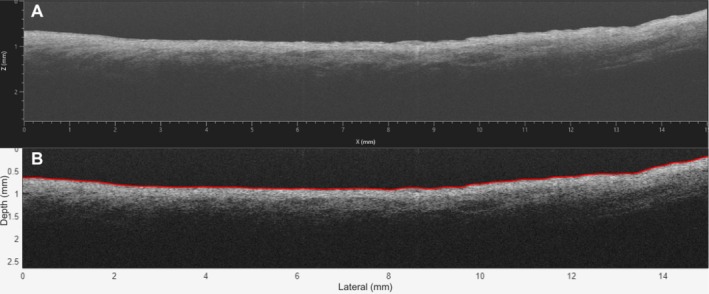
(A) Unaltered optical coherence tomography (OCT) B‐Scan. (B) Image with the detected surface shown in red after MATLAB processing. [Color figure can be viewed in the online issue, which is available at www.laryngoscope.com]

Where the parameters selected for (i) and (ii) are dependent on the OCT system used and were determined by repeated fitting of the surface line on the image. The first depth satisfying both criteria was taken as the surface, and the lateral surface trace was then refined using a median filter followed by Savitzky–Golay smoothing for better display of the detected surface (Figure 2).

The relative attenuation coefficient $\left(\mu_{\mathrm{OCT}}\right)$ was calculated in a region starting at the detected surface and terminating 500 pixels below. We assumed that the VF tissue followed the single scattering model.

$$I\left(z\right)=I_{0}e^{-2\mu_{\mathrm{OCT}}z}$$

where *I*(*z*) is the depth‐dependent intensity, *I*_0_ is the surface intensity, *z* is the detection region (depth), and the factor of two in the exponent accounts for the path length of the light from the source to the interference and detection.

The attenuation was calculated column‐wise by first reversing the machine's automatic log‐corrected A‐scans, and log (dB) to linear unit conversions were conducted as follows:

$$I_{\text{linear}}=10^{\frac{I_{\mathrm{dB}}}{10}}$$

**FIGURE 3:**
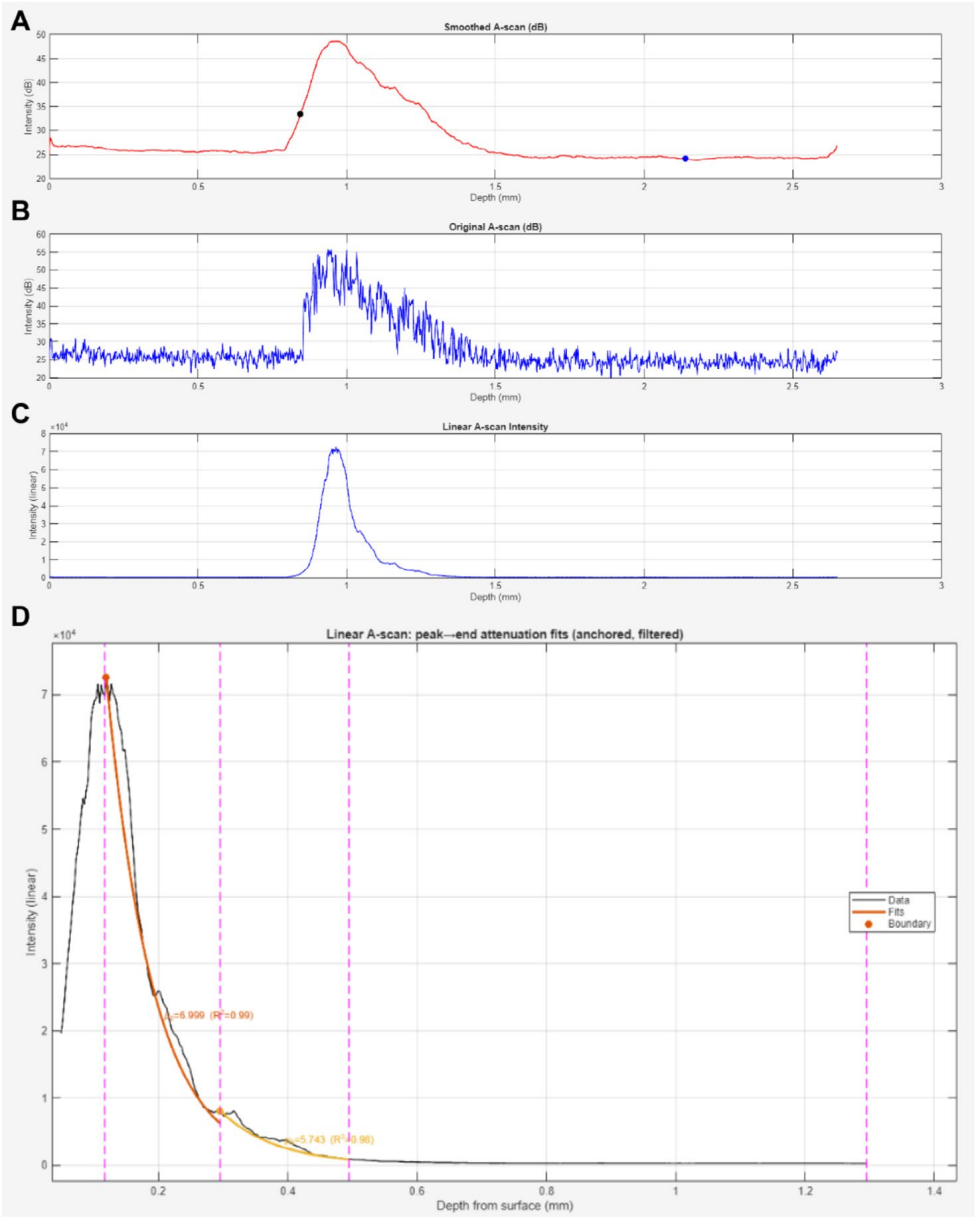
(A) Smoothed decibel signal (log corrected) A‐scan. (B) Raw, unsmoothed A‐scans. (C) Linearized A‐scan intensity profile after reversing the logarithmic correction applied by the OCT system. (D) Linear A‐scan with fitted attenuation regions (orange and yellow) overlaid on the smoothed intensity data (black). Vertical magenta lines indicate valid boundaries used for attenuation calculation. The slope of each fitted region (*R* ^2^ > 0.95) corresponds to the attenuation coefficient (*μ*_OCT_) used in quantitative analysis. Data shown are from a single representative location (pixel 500) of VF3H1 to illustrate the fitting algorithm. [Color figure can be viewed in the online issue, which is available at www.laryngoscope.com]

The linearized A‐scan was then passed through a moving‐average filter to reduce noise and fitted with the MATLAB polyfit function to obtain the relative attenuation along each region of the linear A‐scan. The pixel corresponding to the maximum intensity within each segmented tissue region is used as the anchor point for the attenuation fit. All attenuation calculations were performed from this local intensity maximum to the lower intensity depths within the same region. This approach avoided relying on explicit tissue thickness references. As a result, attenuation was determined directly from the measured optical decay between the local maximum and minimum intensities in each segment. Valid regions were defined as depths with attenuation coefficients below −1.75 mm^−1^ and longer than a threshold distance of 25 pixels: regions of positive slope were omitted from the final calculation. Additionally, valid regions with *R* ^2^ values below 0.95 were discarded to correct for misfits (Figure 3). Following fitting, attenuation coefficients were calculated for each of the 1000 A‐scans across the 15 mm B‐scan and then averaged across all valid regions to report a single representative attenuation coefficient per scan. This averaging approach inherently accounts for spatial variations in tissue microstructure along the VF length. The standard error of attenuation values within each B‐scan was also calculated to quantify intra‐scan spatial variability. This averaged attenuation value represents how changes in hydration affect the bulk tissues' optical properties and is thus a proxy for overall tissue hydration. Attenuation measurements were performed without reference to absolute tissue thickness, using only the local maximum and minimum signal intensities within each segmented region. As a result, the calculated attenuation coefficients are independent of physical tissue dimensions. A fixed analysis depth of 500 pixels (1.295 cm) was selected to fully encompass the VF tissue while excluding the region beneath the fold, which consists of air and therefore exhibits negligible optical attenuation.

### 2.4 Statistical Analysis

All statistical analyses were performed in Jamovi (Version 2.6.44; The Jamovi Project) [27]. A linear mixed‐effects model (LMM) was selected because it appropriately models correlated observations within VFs and continuous within‐sample changes in dehydration. The primary interest was to estimate the linear effect of percent mass loss on attenuation and to test whether this effect differed between sites (interaction term). The dependent variable was the attenuation coefficient. Percent mass loss, a continuous variable representing tissue dehydration, was modeled as a fixed covariate. Site (midline of VFs versus false VFs) was modeled as a categorical fixed factor. To account for repeated measures within larynges, VocalFold ID (each fold) was included as a random intercept. Model fit was summarized using marginal and conditional *R* ^2^ values.

## 3 Results

**FIGURE 4:**
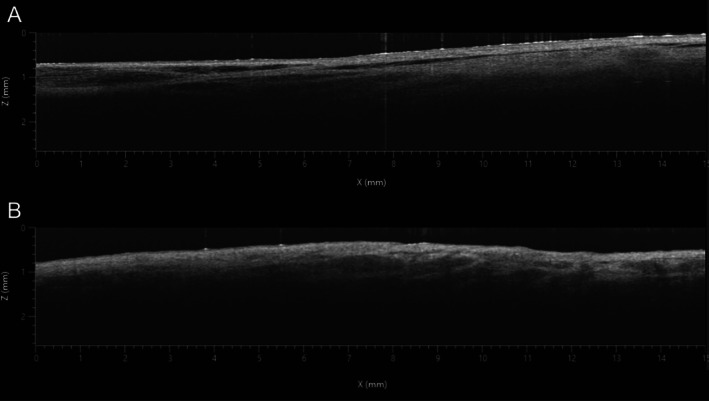
B‐scans of (A) Vocal fold 3 half one (VF3H1) false VFs and (B) VF3H1 true VFs. Tissue depth is the *y*‐axis, and scan length is the *x*‐axis.

B‐Scans of the midline and false folds were visualized in Figure 4. The LMM revealed a significant main effect of percent mass loss on attenuation (*F*(1, 84.6) = 37.79, *p* < 0.001). Across VFs, attenuation decreased by approximately 0.08 mm^−1^ for each 1% increase in tissue mass loss (*b* = −0.079, 95% CI [−0.105, −0.054]). A significant main effect of site was also observed (*F*(1, 81) = 95.03, *p* < 0.001). Midline attenuation values were, on average, 1.47 mm^−1^ higher than false VFs (*b* = 1.468, 95% CI [1.169, 1.767]). The interaction between percent mass loss and site was not significant (*F*(1, 81) = 1.48, *p* = 0.228).

**FIGURE 5:**
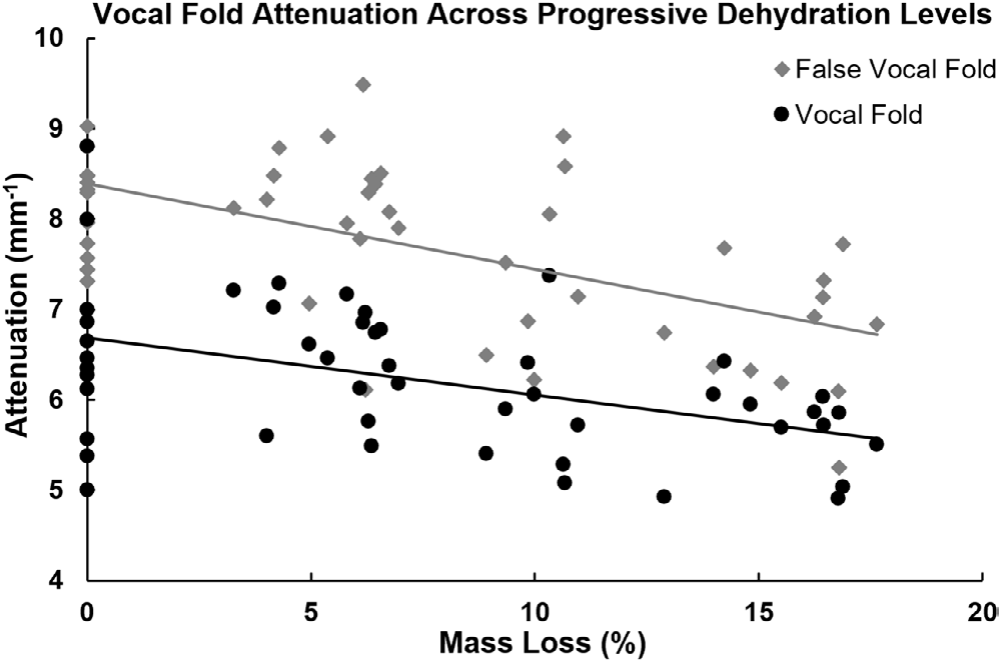
Scatterplot of attenuation versus percent hydration. Diamonds show false vocal fold (VF) scans, while circles show true VF attenuation coefficients at varying hydration levels.

The model explained a substantial proportion of variance in attenuation (marginal *R* ^2^ = 0.576, conditional *R* ^2^ = 0.593). Random intercept variance for VocalFold ID was minimal (ICC = 0.041), suggesting that most of the variability occurred within VFs rather than between VFs. Spatial variability within individual B‐scans was assessed by calculating the within‐scan standard error (SE) of attenuation coefficients across all A‐scans because SE represents the precision of the mean attenuation estimate when all 1000 A‐scans are averaged into a single attenuation coefficient. The SE for all true VF scans averaged to 0.064 and 0.069 mm^−1^ for false VFs. Figure 5 visualizes raw data points for attenuation for all larynges.

## 4 Discussion

To characterize how tissue hydration influences the optical properties of the VFs, the bisected porcine larynges were systematically scanned with OCT across a series of controlled dehydration levels. Each specimen was imaged at baseline and after incremental mass loss to quantify how water reduction alters the OCT‐derived attenuation coefficient. This is the first study to examine attenuation changes with hydration using OCT. The combination of precise mass‐based dehydration control and quantitative OCT analysis established a reproducible framework for linking hydration state to attenuation in VF tissue, providing a foundation for future in vivo research.

The attenuation coefficient reflects how rapidly the OCT signal intensity decays with depth, capturing the combined influence of light scattering and absorption within tissue. Changes in attenuation, therefore, serve as an optical signature of alterations in tissue composition, organization, or water content. Mass loss was shown to be a significant predictor of attenuation changes. This suggests tissue degradation or dehydration reduces light‐tissue interactions, making the tissue less attenuating.

Furthermore, the marginal *R* ^2^ was 0.576, showing a high model fit. While the marginal *R* ^2^ indicates that some variance remains unexplained. While spatial heterogeneity exists within individual VFs, each attenuation coefficient is derived from the mean of 1000 A‐scans, making the SE the relevant precision metric. The mean SE was 0.064 mm^−1^ for the true VFs and 0.069 mm^−1^ for false VFs, and the cumulative attenuation change across the full dehydration range (roughly 1.2 mm^−1^) is approximately 18‐fold larger than any individual scan SE, confirming that spatial heterogeneity does not obscure the dehydration signal. Furthermore, the repeated‐measures design ensures that each specimen serves as its own control across dehydration levels, such that spatial heterogeneity affects all timepoints equally and does not bias the within‐specimen dehydration slope. Additional variance can potentially be attributed to competing effects of absorption and scattering. Dehydration may increase scattering through tissue elements moving closer together [28]; however, absorption is dominant at IR wavelengths [25], which decreases with dehydration. Slight positional variability may also explain the variation. In addition, the conical point‐spread of the OCT beam, in which the initially collimated light diverges into a cone with depth, and the system's sensitivity roll‐off both influence the absolute accuracy of the calculated attenuation coefficient. However, because these effects were identical across all scans, the observed trend with dehydration remains unaffected by the presence of this systematic error. The attenuation variation at baseline reflects biological variability between specimens due to potential differences in tissue microstructure. Critically, while baseline values may vary between specimens, the dehydration response is consistent across VFs. The repeated‐measures design ensures each specimen serves as its own control, such that baseline differences do not bias the within‐specimen dehydration slope.

The negative slope (−0.0794) means that for each 1% increase in mass loss, attenuation decreases by ~0.08 mm^−1^. Results indicated that attenuation decreases linearly with hydration. This aligns with recent work demonstrating that OCT‐derived attenuation coefficients vary with tissue water fraction and, therefore, can serve as quantitative hydration metrics [8]. Furthermore, while both the false and true VF attenuation decreased at a similar rate with hydration, the true VF had a consistent offset of 1.5 mm^−1^ greater than the false VF. This indicates that tissue structure or composition at the true VFs attenuates light more strongly than at the false VFs. Since the attenuation coefficient is a combination of absorbance and scattering, it is important to consider the effects of each. The larynx's underlying microstructure may influence scattering. However, it is difficult to determine whether this difference meaningfully influences attenuation between the false and true VFs because absorption by water dominates at infrared wavelengths. Water molecules possess overtone and combination vibrational modes in this spectral region, meaning that even small changes in tissue hydration or bound‐to‐free water ratio substantially alter light absorption [29, 30]. Given that tissue water content decreased linearly during dehydration, the corresponding linear decline in attenuation coefficient most likely reflects proportional water loss.

The low interfold variability indicates that differences between VFs were minimal, and the main effects of dehydration and site were consistent across larynges with dehydration. This suggests that the relationship between dehydration and attenuation remained consistent across specimens, reinforcing that the observed effects reflect intrinsic tissue optical behavior rather than anatomical or sampling variability. Such reproducibility highlights the robustness of attenuation as a quantitative indicator of VF hydration and supports its potential translation to in vivo applications where intersubject consistency is essential.

Quantifying VF hydration is essential because dehydration has been shown to alter mucosal wave dynamics [31], aerodynamic efficiency [31, 32], tissue stress leading to phototrauma [33], and voice quality [32, 34]. OCT attenuation tracks hydration quantitatively and suggests a pathway toward a noninvasive optical biomarker of VF hydration. In principle, clinicians could monitor hydration or edema dynamics through attenuation changes. Attenuation from OCT has been used clinically to successfully differentiate pediatric anatomy from adults, suggesting attenuation as a metric of VF maturation [18]. Benbouja et al. used OCT for precise inspection of subtle microstructural changes from sulcus, nodules, and cysts, overcoming the insufficient contrast or sensitivity of typical imaging [15]. Coughlan et al. used endoscopic long‐range Doppler OCT to track mucosal wave changes, demonstrating the ability to capture functional vibratory motion of the VFs [9]. While OCT is routinely used in ophthalmology [11, 29] and has been applied clinically to the larynx, the present study is the first investigation to quantify attenuation changes associated with tissue hydration.

An important limitation for clinical translation is that epithelial thickness in humans varies with age and sex, whereas the porcine specimens used in this study were derived from healthy animals of a narrow and consistent age range, yielding comparatively uniform epithelial characteristics [35, 36, 37]. Since our algorithm anchors at the epithelial surface, epithelial thickness variability could affect attenuation measurements. Future human studies should consider alternative anchoring strategies, such as using intensity peaks within the lamina propria or multilayer segmentation to isolate subepithelial hydration changes. Additionally, the OCT machine only uses one wavelength of light. Neubrand et al. found that up to four wavelengths of OCT are required to separate the effects of scattering versus absorption, which is an important consideration for clinical implementation [8]. Only using one wavelength restricts our ability to completely separate absorption from scattering contributions. Future multiwavelength OCT implementations may improve model fit and sensitivity to hydration‐specific optical changes.

Furthermore, the use of ex vivo tissue did not account for some variables that would be present in living tissue. In vivo VFs are perfused, dynamically hydrated, subject to physiologic motion, and bathed in mucosal secretions. The dehydration process here likely reflects uniform bulk water loss rather than localized surface dehydration typical of phonatory stress. However, from a physiological standpoint, phonation dehydrates the VF surface first because it is directly exposed to airflow and mechanical shear [38], so the bulk dehydration model should be roughly representative. The mass‐based dehydration of in situ larynges included minor contributions from adjacent tracheal and cartilaginous tissue rather than VFs alone.

The tissue dehydration and rehydration method could have also introduced variability. Freeze–thaw cycles can disrupt cellular membranes and affect extracellular matrix organization; however, storage in isotonic saline helps preserve tissue structure [39, 40]. VF tissue frozen in saline at −20°C results in minimal changes in viscoelastic properties when proper protocols are followed [40]. Since all larynges underwent identical storage and each served as its own control across progressive dehydration levels, any freezing‐related shift in baseline attenuation would not confound the within‐specimen relationship between mass loss and attenuation. Future validation comparing fresh and frozen tissue could quantify any systematic offset in baseline values. Additionally, examining sequential dehydration and rehydration would help establish OCT's fidelity for assessing bidirectional hydration changes. Validating OCT's ability to monitor reversible hydration changes would therefore showcase hydration as the cause of attenuation changes rather than microstructural differences.

Although percent dehydration values may not precisely reflect changes within the folds themselves, the broad range of mass loss achieved provided a sufficient dynamic window to assess hydration effects, likely exceeding normal physiological variability. Using in situ larynges offers a more anatomically accurate model than isolated VFs, although the masses of individual VFs were not measured separately.

Lastly, since only a single B‐scan was acquired per condition, spatial variability in attenuation or water distribution across the VF could not be assessed. Consequently, it remains unclear which regions of the VF dehydrate most quickly or show the greatest optical change during fluid loss. Future studies should utilize 3D OCT volumetric imaging to map the spatial distribution of attenuation and aim to monitor changes in VF hydration in individual patients undergoing voice‐disorder treatment.

## 5 Conclusion

OCT attenuation provides a quantitative, reproducible measure of dehydration in VFs, which decreased linearly with progressive dehydration of porcine VF tissue. Both Midline and false VFs exhibit similar attenuation declines with tissue mass loss, though with consistent baseline offsets. The minimal interfold variability highlights the reproducibility of OCT‐derived attenuation as a quantitative indicator of hydration. Although the work was performed ex vivo and used a single‐wavelength system, the results provide foundational evidence that attenuation can capture physiologically relevant hydration changes. Translating this approach to in vivo settings could enable clinical noninvasive monitoring of VF hydration, edema, or lesion formation. Findings support further development of OCT as a noninvasive biomarker of VF hydration. Future investigations employing multi‐wavelength OCT, histological correlation, and using 3D OCT will be essential to refine this technique for clinical application.

## Funding

This research was supported by National Institute on Deafness and Other Communication Disorders, NIDCD (grant R01DC015689).

## Conflicts of Interest

The authors declare no conflicts of interest.

## Data Availability

The data that support the findings of this study are available from the corresponding author upon reasonable request.
